# Supplementary material for: The Effect of 2′F-RNA on I-Motif Structure and Stability
Source: Molecules. 2025 Aug 30;30(17):3561. doi: 10.3390/molecules30173561 (PMC12429874; doi:10.3390/molecules30173561)
Supplement: Supplementary file 1 [file molecules-30-03561-s001.zip › molecules-3784098-supplementary.pdf]

SUPPLEMENTARY DATA

The Effect of 2’F-RNA on i-Motif Structure and Stability

Cristina Ugedo [1], Arnau Domínguez [2], Irene Gómez-Pinto, [1] Ramon Eritja, [2,3,*] Carlos González [1,*] and Anna Aviñó [2,3,*]

[1] Instituto de Química Física “Blas Cabrera” (IQF), CSIC, Serrano 119, 28006 Madrid, Spain; cgonzalez@iqf.csic.es (C.G.), cugedo@iqf.csic.es (C.U.), irene_gomez@iqf.csic.es (I.G.P)
[2] Instituto de Química Avanzada de Cataluña (IQAC), CSIC, Jordi Girona 18-26, 08034 Barcelona, Spain; adztnt@cid.csic.es (A.D.), recgma@cid.csic.es (R.E.), anna.avinyo@iqac.csic.es (A.A.)
[3] Centro de Investigación Biomédica en Red de Bioingeniería, Biomateriales y Nanomedicina (CIBER-BBN), 28029 Madrid, Spain.

Supplementary Figures

Figure S1. Exchangeable proton regions of the ^{1}H spectra of FRFull, FR1 and FR2 at different pHs S-2
Figure S2. UV melting profiles. S-2
Figure S3. ^{1}H-NMR and ^{19}F-NMR spectra of FR1 at different temperatures, pH 5. S-3
Figure S4. ^{1}H-NMR FRfull at different temperatures, pH 4. S-3
Figure S5. Native Polyacrylamide gel electrophoresis (PAGE). S-4
Figure S6. Exchangeable protons region of the NOESY spectra and ^{19}F-^{1}H HOESY spectra of FR1. S-4
Figure S7. Ensemble and average structures of FR1. S-5
Figure S8. Ensemble and average structures of FR2. S-6
Figure S9. Mass spectrometry spectrum of FR1. S-7
Figure S10. Mass spectrometry spectrum of FR2. S-7
Figure S11. Mass spectrometry spectrum of FRFull. S-8

Supplementary Tables

Table S1. Chemical shifts of FR1. S-9
Table S2. Chemical shifts of FR2. S-9
Table S*3*. Experimental constraints and calculation statistics of FR1 and FR2. S-10
T**able S4.** Deoxyribose conformations of FR1 and FR2 according to J-coupling data S-11
Table S*5*. Average dihedral angles and order parameters of the structure of FR1. S-12
Table S*6*. Average dihedral angles and order parameters of the structure of FR2. S-13
Table S*7*. Oligonucleotide characterization. S-14

Supplementary Figures

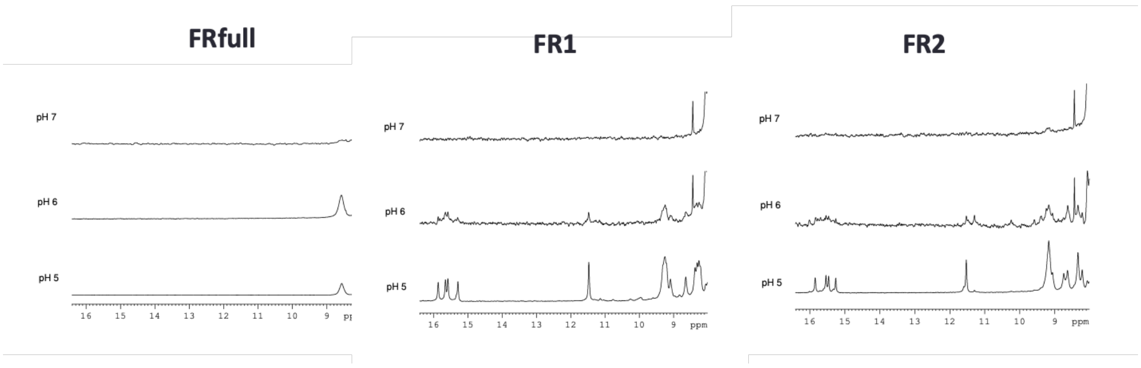

Figure S1. Exchangeable proton regions of the ^{1}H spectra of FRFull, FR1 and FR2 at different pHs (25 mM sodium phosphate, T= 5ºC).

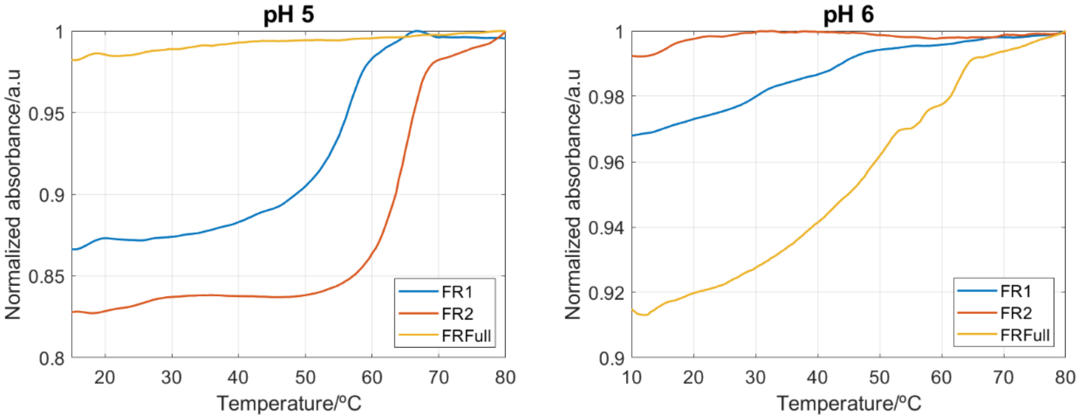

Figure S2. UV melting profiles at two different pH values (25 mM sodium phosphate buffer).

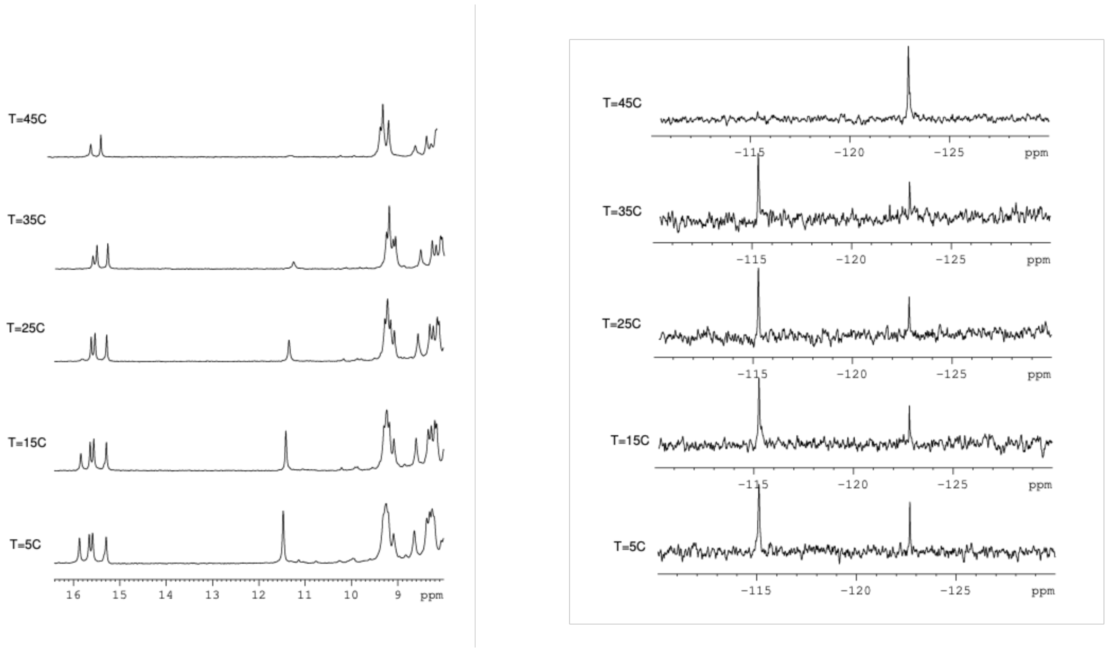

Figure S3. Left) ^{1}H-NMR (exchangeable protons region), and Right) ^{19}F-NMR spectra of FR1 at different temperatures (25 mM sodium phosphate, pH 5).

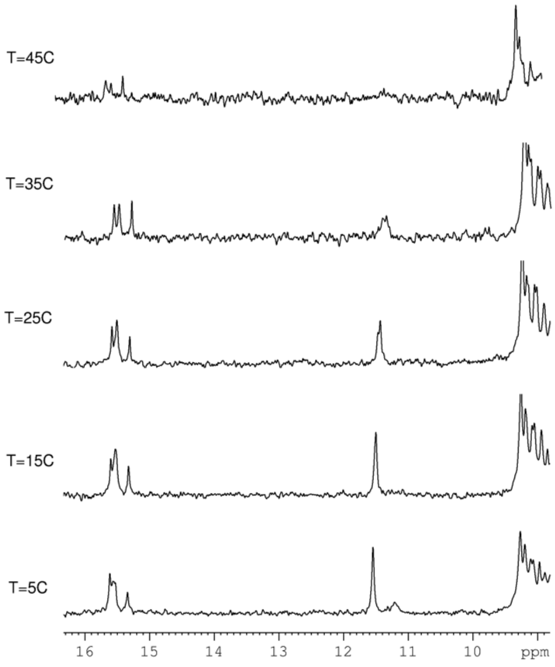

Figure S4. ^{1}H-NMR (exchangeable protons region), FRfull at different temperatures (25 mM sodium phosphate, pH 4)

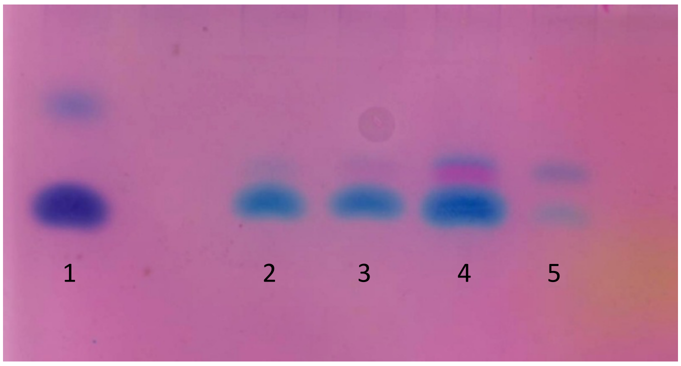

Figure S5.- 12% Native Polyacrylamide gel electrophoresis (PAGE), pH 6.0, 100 μM strand concentration. Lane 1: Bromophenol blue/Xylene Cyanol 1:1, Lane 2: Control unmodified dTC$_5$, Lane 3: FR1, Lane 4: FR2 and Lane 5: FRFull.

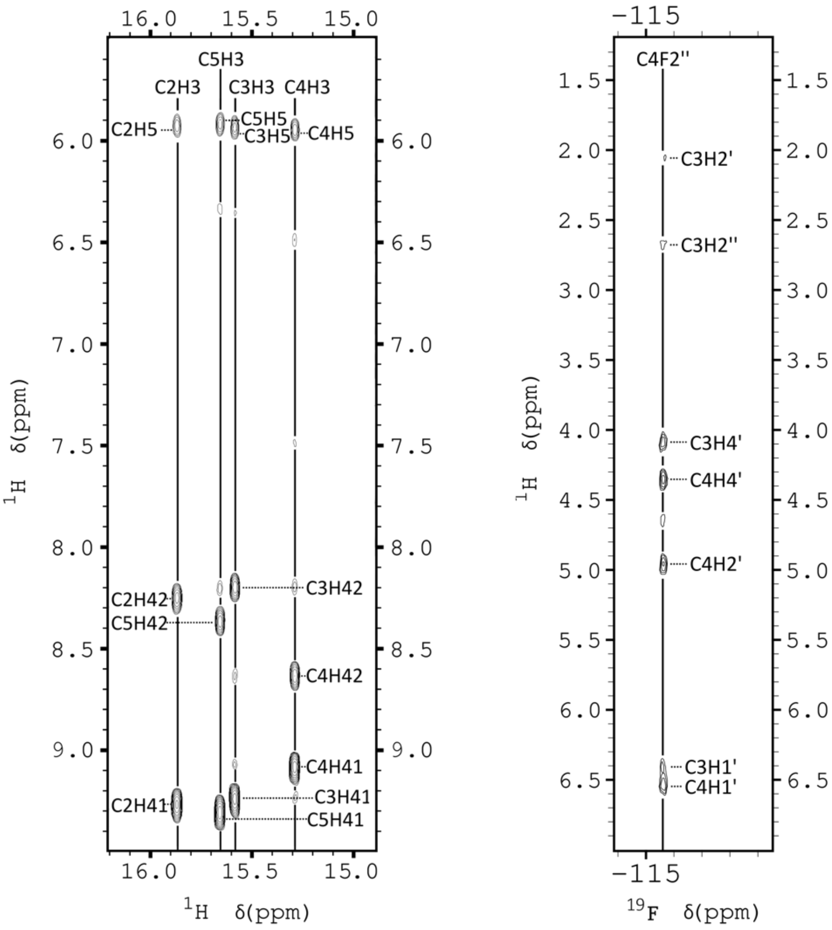

Figure S6. Left) Exchangeable protons region of the NOESY spectra of FR1, showing imino-amino cross-peaks. Right) ^{19}F-^{1}H HOESY spectra of FR1, indicating the most relevant ^{19}F-^{1}H cross-peaks. (25 mM sodium phosphate, pH 5, T= 5°C).

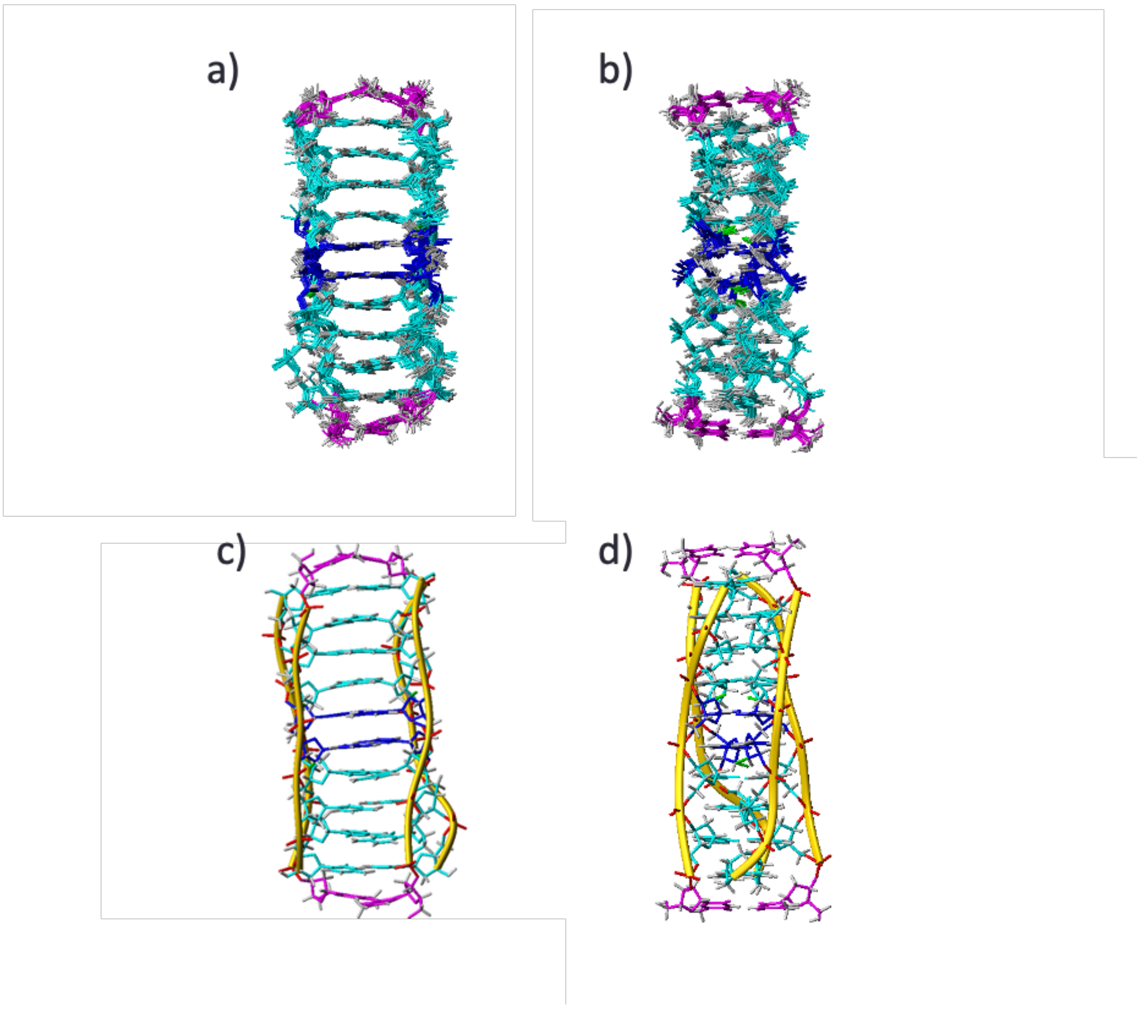

Figure S7.- Two views of the ensemble (a, b), and average (c,d) of the resulting structures of FR1. Views a,c are from the major groove; b,d from the minor groove. Color code: blue, 2'F-araC; cyan, dG; magenta dT.

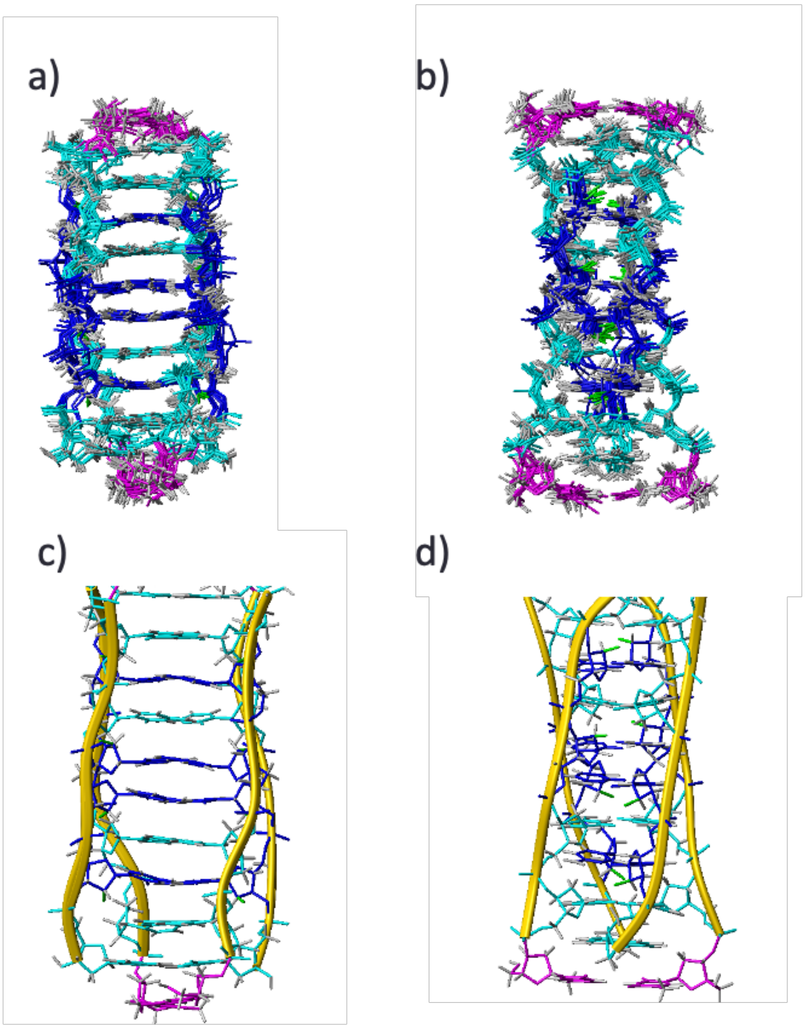

Figure S8.- Two views of the ensemble (a, b), and average (c,d) of the resulting structures of FR2. Views a,c are from the major groove; b,d from the minor groove. Color code: blue, 2'F-araC; cyan, dG; magenta dT.

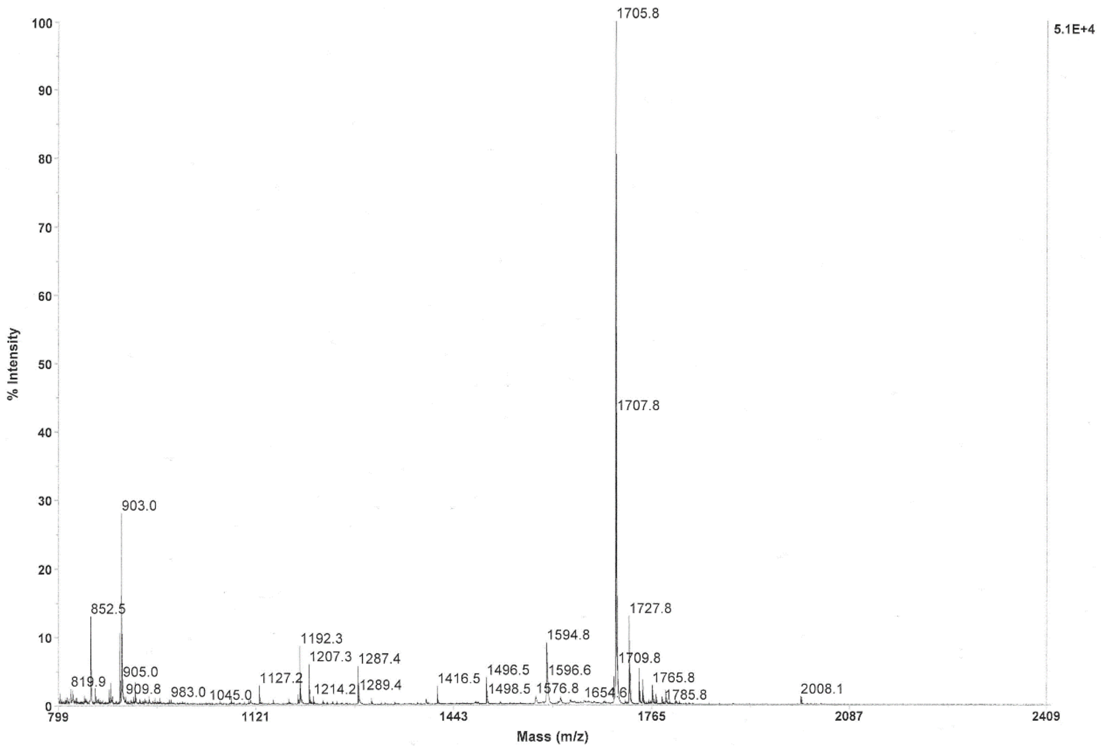

Figure S9. Mass spectrometry spectrum of FR1 oligonucleotide.

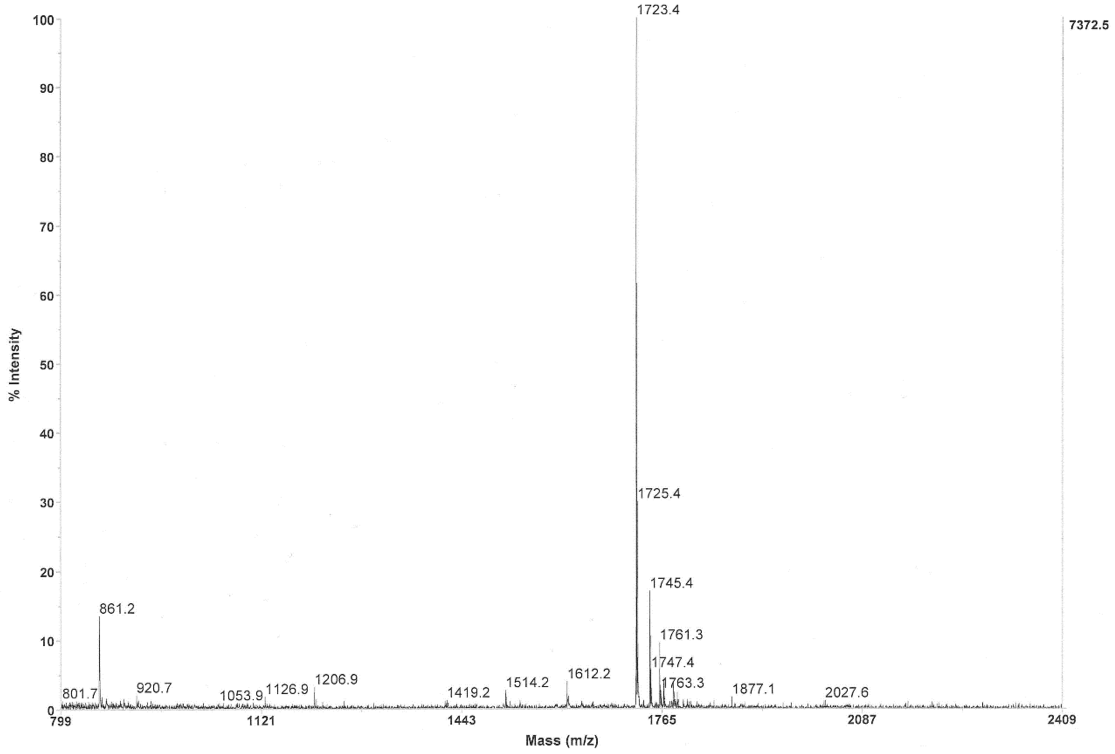

Figure S10. Mass spectrometry spectrum of FR2 oligonucleotide.

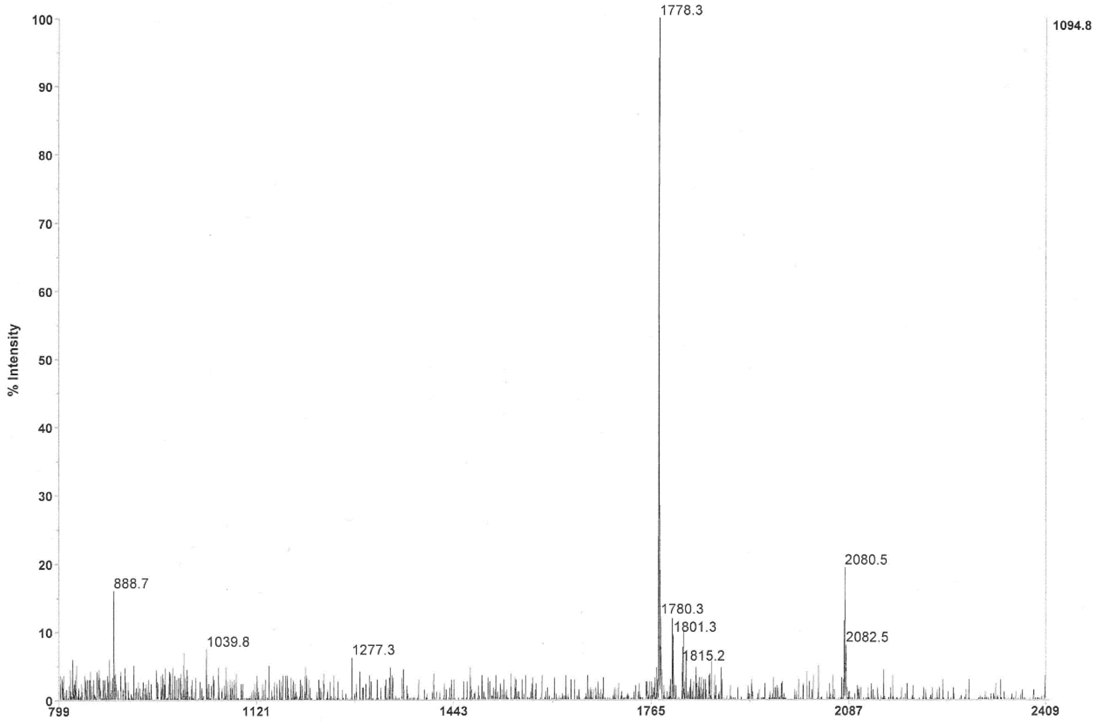

Figure S11. Mass spectrometry spectrum of FRFull oligonucleotide.

Supplementary Tables

Table S1.- Chemical shifts of FR1, pH 5, T=5ºC

	H1/H3/H3$^+$	H42/H22	H41/H21	H6/H8	H5/Me	H1’	H2’	H2”F2’’	H3’
T1	11.47	-	-	7.65	1.77	6.34	2.64	2.18	4.84
C2	15.87	8.25	9.27	7.92	5.94	6.48	2.08	2.51	4.85
C3	15.58	8.21	9.24	7.68	5.96	6.38	2.02	2.67	4.82
C4	15.29	8.64	9.09	7.50	5.96	6.52	4.93	-115.12	4.63
C5	15.66	8.37	9.32	7.61	5.92	6.35	2.16	2.58	4.65
C6	15.65	8.31	9.20	7.74	5.97	6.19	2.20	2.58	4.30

n.o.: not observed
n.a.: not assigned

Table S2.- Chemical shifts of FR2, pH 5, T=5ºC

	H1/H3/H3$^+$	H42/H22	H41/H21	H6/H8	H5/Me	H1’	H2’	H2”F2’’	H3’
**T1**	11.52	-	-	7.64	1.79	6.33	2.61	2.14	4.83
**C2**	15.84	8.20	9.15	7.76	6.02	6.35	2.58	1.98	4.86
**C3**	15.53	8.32	9.19	7.67	6.01	6.39	2.01	2.62	4.86
**C4**	15.25	8.73	9.05	7.53	6.00	6.53	4.87	-115.32	4.67
**C5**	15.46	8.62	9.13	7.50	5.96	6.52	5.10	-116.82	4.64
**C6**	n.o.	8.33	9.15	7.73	6.02	6.29	2.41	2.16	4.34

n.o.: not observed
n.a.: not assigned

Table S3.- Experimental constraints and calculation statistics of FR1 and FR2.

	FR1		FR2	
**Experimental distance constraints**				
Total number	274		300	
Intra-residue	0		0	
sequential	116		128	
Intra-strand	20		4	
Inter-strand	110		132	
F—H constraints	28		36	
**RMSD (Å)**				
all bases	0.5 ± 0.2		0.4 ± 0.1	
backbone	0.8 ± 0.1		0.7 ± 0.1	
all heavy atoms	0.7 ± 0.1		0.6 ± 0.1	
Residual violations	Average	Range	Average	Range
Sum of violation (Å)	3.13	2.77… 3.85	3.13	2.40 … 3.75
Max. violation (Å)	0.43	0.38 … 0.47	0.29	0.21 … 0.35
NOE energy[#] (kcal/mol)	10.96	3.37 … 16.87	10.06	3.36 … 17.29
Total energy (kcal/mol)	- 5008.7	- 3914.8 … - 5238.6	- 5322.4	- 2925.0 … - 5777.8

[*] All except thymines 5,10,11,12,13,18

[#] K_{NOE} = 20 kcal/(mol·Å^2)

[*] All except thymines 11,12

Table S4.- Deoxyribose conformations of FR1 and FR2 according to J-coupling data.

Residue	Sugar conformation	
	FR1	FR2
T1	South	South
C2	North	North
C3	North	North
C4	North	North
C5	North	North
C6	North	North

Table S5.- Average dihedral angles and order parameters of the structure of FR1.

Residue	Pseudorot.		α		β		γ		δ		ε		ζ		χ	
	Phase	Ampl.	Ave.	OP	Ave.	OP	Ave.	OP	Ave.	OP	Ave.	OP	Ave.	OP	Ave.	OP
**T1**	147	48					-2	0.5	142	1	-95	0.9			-126	1
**C2**	27	52	69	1	-148	1	-176	1.0	70	1	-175	1	-102	1	-136	1
**C3**	3	38	-57	1	-177	1	70	1.0	93	1	178	1	-109	1	-114	1
**C4**	7	40	-72	1	177	1	78	1.0	82	1	-169	1	-105	1	-128	1
**C5**	7	26	-53	1.0	176	1	75	1.0	98	1	71	1	-96	1	-123	1
**C6**	46	42	69	0.6	173	1	-164	1.0	78	1			79	1	-115	1
**T7**	157	45					-21	0.5	146	1	-127	0.8			-123	1
**C8**	42	47	95	1.0	-176	1	-170	1.0	73	1	-172	1	-84	0.9	-116	1
**C9**	30	41	-68	0.9	-172	1	62	1.0	80	1	175	1	-87	1.0	-104	1
**C10**	21	42	-56	0.8	171	1	69	0.9	72	1	-159	1	-89	1	-122	1
**C11**	37	43	-63	0.7	-178	1	67	0.9	77	1	-177	1	-78	1	-112	1
**C12**	43	44	-59	0.8	178	1	69	1.0	77	1			-81	1	-117	1
**T13**	65	21					59	0.7	103	1	-173	0.8			-118	1
**C14**	7	35	-72	0.2	-171	1	64	1.0	88	1	-165	1	-94	1	-123	1
**C15**	35	39	-66	0.7	-173	1	64	1.0	83	1	178	1	-89	1	-103	1
**C16**	23	45	-60	1.0	170	1	69	1.0	71	1	-165	1	-90	1	-123	1
**C17**	22	46	-68	1.0	-172	1	70	1.0	73	1	179	1	-77	1	-117	1
**C18**	32	32	39	0.8	173	1	-169	1.0	75	1			69	1	-111	1
**T19**	65	29				1	28	0.5	99	1	-168	0.9			-121	1
**C20**	5	41	-77	0.5	175	1	70	1.0	86	1	-178	1	-106	1	-140	1
**C21**	14	34	-72	1.0	-152	1	71	1.0	94	1	177	1	-87	1	-107	1
**C22**	6	42	-62	1.0	168	1	74	1.0	76	1	-161	1	-95	1	-128	1
**C23**	9	40	180		-138	0.8	170		82		-175	1	-116	0.8	-134	1
**C24**	15	23	60	0.6	164	1	-154	1.0	76	1			79	1	-110	1

Table S6.- Average dihedral angles and order parameters of the structure of FR2.

Residue	Pseudorot.		α		β		Y		δ		ε		ζ		X	
	Phase	Ampl.	Ave.	OP	Ave.	OP	Ave.	OP	Ave.	OP	Ave.	OP	Ave.	OP	Ave.	OP
**T1**	161	39					57	0.8	143	1	-132	1.0			-143	1.0
**C2**	15	44	89	1.0	-171	1.0	-174	1.0	71	1	173	1.0	-69	1.0	-133	1.0
**C3**	6	38	-64	1.0	-171	1.0	73	1.0	89	1	-170	1.0	-92	1.0	-127	1.0
**C4**	19	40	-125	0.7	-174	1.0	126	0.7	77	1	-170	1.0	-87	1.0	-134	1.0
**C5**	54	21	172	0.5	-126	0.6	167	0.9	80	1	-174	1.0	113	0.9	-150	1.0
**C6**	39	38	-70	0.6	-141	1.0	66	1.0	88	1			-78	1.0	-108	1.0
**T7**	151	42					57	0.8	96	0.9	-160	1.0			-133	0.9
**C8**	28	47	-75	1.0	-169	1.0	57	1.0	77	1	179	1.0	-82	1.0	-143	1.0
**C9**	3	42	-55	0.9	-175	1.0	77	1.0	92	1	-174	1.0	-93	1.0	-124	1.0
**C10**	7	42	-140	0.6	-156	0.9	131	0.7	79	1	-166	1.0	-94	0.8	-135	1.0
**C11**	14	36	171	0.7	-136	1.0	166	0.9	74	1	179	0.9	-108	0.9	-148	0.9
**C12**	4	31	-61	0.8	-152	1.0	75	1.0	97	1			-84	0.7	-108	0.9
**T13**	149	43					60	0.8	117	0.9	-138	0.9			-136	0.9
**C14**	27	34	-90	0.2	171	0.9	70	1.0	86	1	171	1.0	-93	1.0	-143	1.0
**C15**	8	39	-62	0.7	-151	1.0	72	1.0	111	1	-157	0.9	-88	1.0	-107	1.0
**C16**	16	43	-88	1.0	156	0.6	108	0.7	72	1	-168	1.0	-80	1.0	-134	1.0
**C17**	5	38	-64	1.0	-173	1.0	68	0.9	76	1	-177	0.9	-79	1.0	-129	1.0
**C18**	31	26	-55	0.6	-176	0.8	64	1.0	88	1			-87	0.8	-112	1.0
**T19**	124	29					51	0.8	110	0.9	-134	0.9			-145	0.9
**C20**	31	43	-85	0.5	166	1.0	66	1.0	85	1	172	1.0	-98	1.0	-145	1.0
**C21**	13	40	-66	1.0	-149	0.9	77	1.0	105	1	-167	1.0	-87	1.0	-108	1.0
**C22**	13	42	-104	0.7	156	0.7	120	0.7	73	1	-170	1.0	-75	1.0	-134	1.0
**C23**	10	37	-168	0.8	-124	0.9	151	0.9	143	1	177	1.0	108	1.0	-144	1.0
**C24**	18	29	-62	1.0	-153	1.0	73	1.0	71	1			-86	1.0	-102	0.9

Table S7. Oligonucleotide characterization. HPLC conditions: The buffers used for the HPLC analysis were Buffer A: 5 % acetonitrile in 0.1 M of triethylammonium acetate (TEAAc) and Buffer B: 70 % acetonitrile in 0.1 M of triethylammonium acetate (TEAAc). HPLC was performed on a Waters chromatography system with a Waters 2996 Photodiode Array detector using an XBridg^{TM}OST C18 – 2.5 µm (10x50mm) column. The prepared oligonucleotides were analyzed using a gradient from 0 to 20 % buffer B in 20 min.

Name	MW Calc (g·mol-1)	MW Exp (g·mol-1)	r.t (min)
FR1	1705.3	1705.8	10.1
FR2	1723.3	1723.4	11.2
FRFull	1777.3	1778.3	12.9
